# Metabolomic Profiling of Middle Ear Effusion Suggests a Predominant Influence of Age over Viscosity: An HR-MAS NMR Study

**DOI:** 10.3390/ijms27010020

**Published:** 2025-12-19

**Authors:** Seokhwan Lee, Seonghye Kim, Sojeon Moon, Se-Joon Oh, Seok-Hyun Kim, Hyun-Min Lee, Suhkmann Kim, Sung-Won Choi

**Affiliations:** 1 Department of Otorhinolaryngology, Biomedical Research Institute, Pusan National University Hospital, Busan 49241, Republic of Korea; entlsh85@gmail.com (S.L.); entmania@pusan.ac.kr (S.-J.O.); 2 Department of Chemistry and Chemistry Institute for Functional Materials, Pusan National University, Busan 43241, Republic of Korea; seonghyeee@pusan.ac.kr (S.K.); sojeon@pusan.ac.kr (S.M.); 3 Department of Otorhinolaryngology-Head and Neck Surgery, Pusan National University School of Medicine, Busan 49241, Republic of Korea; hmlee01@hanmail.net; 4 Department of Otorhinolaryngology, Biomedical Research Institute, Pusan National University Yangsan Hospital, Yangsan 50612, Republic of Korea; bluekoiz@naver.com

**Keywords:** otitis media with effusion, metabolomics, mucins, viscosity, middle ear

## Abstract

Otitis media with effusion (OME) involves heterogeneous middle ear effusion (MEE), and its classification based on viscosity (serous/mucous) is often confounded by patient age. This study determined the independent contributions of age and viscosity to the MEE metabolome. In this prospective study, high-resolution magic-angle spinning nuclear magnetic resonance analysis of 83 MEE samples (45 adult serous, 17 child serous < 12 years, and 21 child mucous) was performed. Statistical analyses included principal component analysis, correlation analysis, analysis of covariance (ANCOVA), and age-adjusted receiver operating characteristic curve analysis. Age was the primary metabolic variance determinant, overshadowing viscosity-related differences. Significant age-associated metabolic trends were identified: pyruvate and lactate levels increased with age, whereas glutamate and leucine levels decreased, indicating energy and inflammatory metabolism shifts. After adjusting for age using ANCOVA, taurine, glycine, and choline were significantly associated with effusion viscosity, and a combined panel of these metabolites achieved an age-adjusted area under the curve of 0.707 (95% confidence interval: 0.55–0.89). In conclusion, the MEE metabolic profile was more strongly influenced by patient age than by viscosity, suggesting fundamental differences in OME pathophysiology between children and adults. Nonetheless, specific viscosity-associated metabolites were identified, offering a basis for objective metabolic typing.

## 1. Introduction

Otitis media with effusion (OME) is defined by chronic fluid presence in the middle ear cavity behind an intact tympanic membrane, without acute infection signs [1]. It is a pervasive condition, particularly in the pediatric population, where it is a leading cause of acquired hearing impairment and frequently requires surgical intervention, including ventilation tube insertion [2]. Although OME affects individuals throughout their lifespans, its presentation and underlying mechanisms vary significantly between children and adults [3,4].

A critical OME heterogeneity characteristic is middle ear effusion (MEE), which is clinically categorized into serous (thin and watery) or mucous (thick and tenacious) based on its viscosity [5]. Notably, this distinction is not merely descriptive; mucous effusion is often associated with prolonged disease duration, increased recurrence risk, and potentially worse audiologic outcomes than serous effusion [6,7].

The distinct physical properties of these effusions were attributed to their molecular differences. Substantial research has established that mucous effusions exhibit significantly higher levels of mucin glycoproteins, particularly MUC5B, the primary high viscosity determinant [6]. Furthermore, studies have reported differential expression of inflammatory mediators and distinct cytological profiles, often suggesting a more robust inflammatory response in mucous MEE [8,9,10]. Given these clear molecular distinctions in mucin and inflammatory markers, the overall metabolic landscape may also differ significantly based on effusion viscosity.

Nevertheless, interpreting these differences is complicated by a major clinical observation, namely, the strong correlation between patient age and effusion type. Mucous effusions are prevalent in young children, whereas serous effusions are more common in adults [5,11]. OME in children is frequently associated with developmental factors such as anatomical immaturity of the eustachian tube and a developing immune system prone to frequent upper respiratory infections, which foster chronic inflammation and mucus hypersecretion [5,12]. Conversely, adult-onset OME may have distinct etiologies, including persistent inflammation, allergies, and structural abnormalities [11].

This strong association raises a critical question: are the observed molecular and cellular differences truly attributable to the effusion phenotype (viscosity) or are they secondary manifestations of the host’s age and associated immunological status? Importantly, most previous studies have not adequately controlled for this confounding effect.

Therefore, a system-level approach is required to gain a comprehensive understanding of the OME biochemical environment. Metabolomics, the systematic analysis of small-molecule metabolites, provides a functional readout of cellular activity and the physiological state of biological systems [13]. Notably, analyzing the MEE metabolome captured the metabolic activities of host epithelial cells, infiltrating immune cells, and the local microenvironment [14]. Furthermore, high-resolution magic-angle spinning nuclear magnetic resonance (HR-MAS NMR) spectroscopy has demonstrated effectiveness for analyzing heterogeneous and semi-solid biological samples [15]. Because middle ear effusion ranges from low-viscosity serous fluid to highly viscous, gel-like mucoid material, HR-MAS can be advantageous, particularly for mucoid MEEs, by enabling metabolic profiling with minimal sample preparation [15].

This study aimed to characterize the MEE profiles of children and adults with different effusion types using HR-MAS NMR-based metabolomics. To the best of our knowledge, this represents the first systematic attempt to dissect the independent influences of age and viscosity on the MEE metabolome and to identify age-adjusted metabolic biomarkers.

## 2. Results

### 2.1. Global Metabolomic Profiling of Middle Ear Effusions

**Figure 1:**
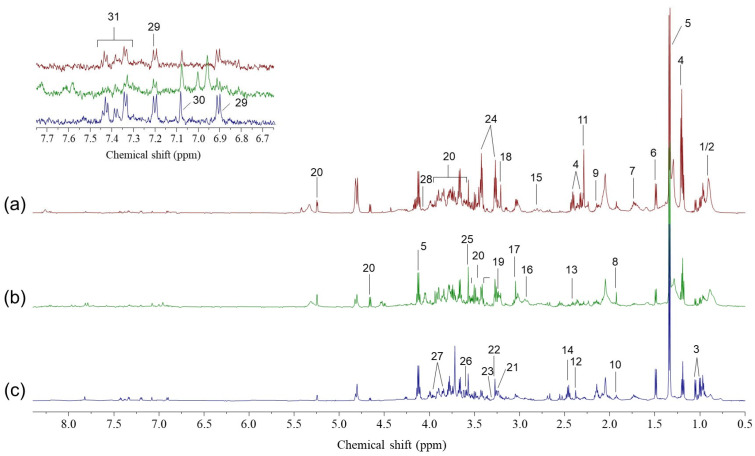
Representative 600 MHz HR-MAS ^1^H NMR spectra of middle ear effusion samples. (**a**) Mucous effusion from a child (Mucous_Child), (**b**) serous effusion from a child (Serous_Child), and (**c**) serous effusion from an adult (Serous_Adult). The inset displays an expanded aromatic region (7.7–6.7 ppm). Peak assignments: 1, isoleucine; 2, leucine; 3, valine; 4, 3-hydroxybutyrate; 5, lactate; 6, alanine; 7, lysine; 8, acetate; 9, glutamate; 10, proline; 11, acetoacetate; 12, pyruvate; 13, succinate; 14, glutamine; 15, citrate; 16, asparagine; 17, creatine; 18, choline; 19, sn-glycerol-3-phosphocholine; 20, glucose; 21, arginine; 22, betaine; 23, myo-inositol; 24, taurine; 25, glycine; 26, threonine; 27, serine; 28, cystine; 29, tyrosine; 30, histidine; 31, phenylalanine.

HR-MAS ^1^H-NMR spectroscopy was used to analyze the MEE sample metabolic composition. Representative spectra from the Serous_Adult (SA), Serous_Child (SC), and Mucous_Child (MC) groups are shown in Figure 1. Overall, 31 metabolites were identified and quantified, including amino acids, carboxylic acids, carbohydrates, amines, and other organic compounds (Supplementary Table S1).

**Figure 2:**
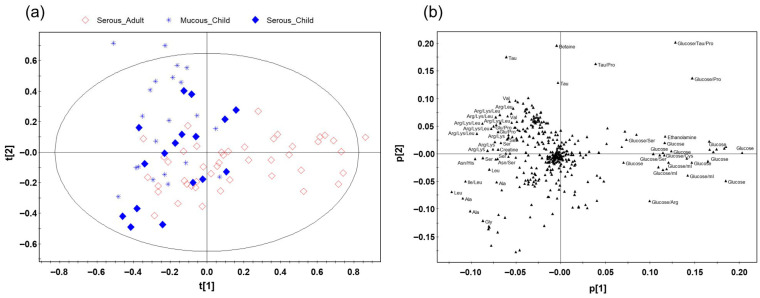
Principal component analysis of HR-MAS ^1^H NMR spectra from middle ear effusion samples (R^2^X = 0.859, Q^2^ = 0.482). (**a**) Score plot demonstrating sample distribution among the three groups, including Serous_Adult (red diamonds), Serous_Child (blue squares), and Mucous_Child (light-blue asterisks). (**b**) Corresponding loading plot illustrating the metabolites responsible for group separation. Ala, alanine; Arg, arginine; Cys, cysteine; Gln, glutamine; Glu, glutamate; Gly, glycine; His, histidine; Ile, isoleucine; Lac, lactate; Leu, leucine; Lys, lysine; Pro, proline; Ser, serine; Tau, taurine; Val, valine.

To investigate the overall metabolic patterns associated with age and effusion type, unsupervised PCA was performed on the normalized spectral data. The PCA score plot (Figure 2) revealed a striking separation between the adult (SA) group and the two children groups (SC and MC), demonstrating that age is the predominant factor driving MEE metabolic variation. Within the children cluster, only partial segregation was observed between the serous and mucous types, indicating that their overall metabolic profiles were relatively similar compared to the profound differences observed between children and adults.

### 2.2. Differential Metabolites Identified Among Effusion Groups

We performed a one-way ANOVA to identify the specific metabolites that demonstrated significant variation among the three groups. Seventeen metabolites exhibited significant differences (FDR < 0.05) (Table 1, Supplementary Table S2).

Fisher’s least significant difference post hoc analysis helped delineate whether these differences were primarily driven by age or effusion type. Most metabolites exhibited significant differences between adults and children (SA vs. SC and SA vs. MC), but not between the two child groups (SC vs. MC). These age-related metabolites included pyruvate, glutamate, citrate, lactate, asparagine, cysteine, glutamine, lysine, leucine, glucose, betaine, and sn-Glycero-3-phosphocholine.

The metabolites primarily associated with effusion type (significant difference between MC and SC) were taurine and succinate. Glycine and choline levels exhibited significant differences across all three pairwise comparisons, suggesting an influence of age and type.

### 2.3. Age-Dependent Metabolic Variations Dominate the MEE Profile

PCA and ANOVA results strongly indicated that patient age was the primary MEE metabolome determinant. To isolate the effect of age on viscosity, we performed Spearman correlation analysis using only serous samples (SA + SC).

These results confirmed significant age-dependent metabolic shifts (Table 1 and Supplementary Table S3). Specifically, pyruvate exhibited the strongest positive correlation with age (r = 0.603, FDR < 0.001), indicating a marked increase in its concentration with age. Similarly, lactate (r = 0.371), asparagine (r = 0.394), and betaine (r = 0.365) levels were significantly and positively correlated.

Conversely, several metabolites were significantly negatively correlated with age. Glutamate (r = −0.433, FDR < 0.01), leucine (r = −0.381), and creatine (r = −0.363) concentrations were significantly lower in adults than in children. These findings highlight the profound age-related modulation of energy metabolism (pyruvate, lactate) and amino acid metabolism (glutamate, leucine) in the middle ear microenvironment.

### 2.4. Identification of Viscosity Type-Dependent Metabolites After Adjusting for Age

Because age dominated the overall metabolic profiles, potentially masking subtle differences in viscosity, we employed ANCOVA to identify metabolites associated with effusion type while controlling for age as a covariate.

The ANCOVA results confirmed that several metabolites remained significantly different between the mucous and serous effusions, even after age adjustment (Table 1 and Supplementary Table S4). Taurine (FDR = 0.0092), glycine (FDR = 0.0139), choline (FDR = 0.0398), and citrate (FDR = 0.0422) were identified as key viscosity-dependent indicators, independent of age.

### 2.5. Discriminatory Performance of Viscosity-Specific Biomarkers

We evaluated the potential of the identified metabolites (taurine, glycine, choline) to distinguish between mucous and serous effusions using an AROC analysis, which accounted for the confounding effects of age.

**Figure 3:**
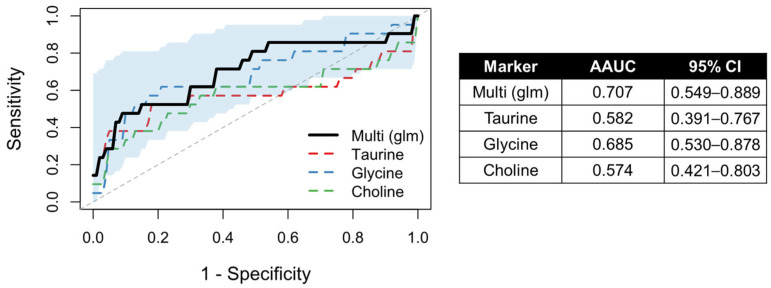
Covariate-adjusted ROC curves for distinguishing mucous-type from serous-type middle ear effusions. Individual metabolites (taurine, glycine, and choline) were evaluated after age adjustment (dashed lines), and their combined discriminative performance was assessed using a multi-adjusted logistic model (Multi glm, solid black line). The shaded area represents the 95% confidence interval of the multi-metabolite model.

The individual age-adjusted areas under the curve (AAUCs) were modest for glycine (AAUC = 0.685), taurine (AAUC = 0.582), and choline (AAUC = 0.574), indicating limited discriminative power when considered individually (Figure 3 and Supplementary Table S5).

Nevertheless, a multivariate AROC model combining these three metabolites (glycine + taurine + choline) achieved a higher discriminative performance, with an AAUC of 0.707 (95% CI = 0.55–0.89) (Figure 3). Therefore, combining these three metabolites can distinguish mucus from serous effusions more reliably compared to the individual markers, independent of patient age.

## 3. Discussion

This study aimed to characterize the metabolic landscape of MEE in OME. Based on the established literature highlighting distinct molecular profiles related to mucin expression and cytology, we hypothesized that effusion viscosity (serous vs. mucous) would be the primary metabolic profile determinant [5,6,8,9,10]. Nonetheless, comprehensive metabolomic profiling conducted using HR-MAS NMR demonstrated that the patient’s age exerted a far greater influence on the overall MEE metabolome compared to the physical characteristics of the effusion, thereby suggesting fundamental differences in the underlying pathophysiology and inflammatory metabolism of OME in children and adults.

The specific metabolic shifts observed provide insight into these age-related differences, particularly regarding energy and amino acid utilization. We found a strong positive correlation between age and pyruvate and lactate levels, which are key glycolysis metabolites [16,17]. Notably, elevated levels in adults suggest a heightened glycolytic rate in the middle ear [18]. This metabolic reprogramming towards glycolysis, even in the presence of oxygen (the Warburg effect), is a hallmark of activated immune cells during inflammation, providing the necessary energy and biosynthetic precursors to sustain the inflammatory response [19,20]. Alternatively, increased lactate levels may indicate a more hypoxic environment in the adult middle ear cavity than in children [9,21].

Conversely, the concentrations of several amino acids, notably glutamate and leucine, were significantly higher in children than in adults, strongly suggesting a more metabolically active and anabolic host response in pediatric patients. Specifically, higher leucine levels can directly enhance immune cell proliferation and tissue remodeling by activating the mTOR pathway [22,23,24]. Concurrently, the abundance of glutamate likely supports these high-energy demands by replenishing the tricarboxylic acid cycle and bolstering antioxidant defenses [25,26]. This coupling of key amino acids with fundamental anabolic and energetic pathways highlights that the pediatric inflammatory response is intrinsically linked to developmental processes, marking a distinct metabolic signature compared to that in adults.

Although the overall metabolic profiles were dominated by age, we successfully identified metabolites specifically associated with effusion viscosity using rigorous ANCOVA. Taurine, glycine, and choline have emerged as robust, age-independent markers that differentiate mucous from serous effusions. These metabolites are functionally linked to processes critical to the physical properties of the effusion.

Taurine is among the most abundant free amino acids in mammals and is crucial in osmoregulation, antioxidant defense, and inflammation modulation [27,28,29]. Moreover, the accumulation of high molecular weight mucins in mucous effusion significantly alters the osmotic environment of the middle ear, creating conditions impairing normal fluid clearance mechanisms [6,30]. The significant taurine level differences may reflect cellular osmoregulatory responses to the altered osmotic environment or compensatory fluid transport mechanism changes required to maintain cellular homeostasis in this viscous environment [28,31].

The interpretation of glycine differences is related to both mucin synthesis pathways and the inflammatory redox state. The peptide backbone of mucin glycoproteins is characterized by tandem repeats rich in serine, threonine, and proline, which represent primary sites of extensive O-glycosylation [32]. Glycine is readily interconverted with serine via serine hydroxymethyltransferase [33]. Thus, altered glycine levels may reflect an increased metabolic demand for serine during the enhanced synthesis of mucin glycoproteins, such as MUC5B, which is markedly overexpressed in mucoid middle ear effusions [34]. Furthermore, glycine is an essential precursor in the synthesis of glutathione (GSH), a major endogenous antioxidant tripeptide [35]. Given the oxidative stress associated with chronic inflammation, free glycine level differences may also reflect altered antioxidant capacity or GSH turnover rates in the inflamed middle ear environment [35].

Choline is essential for phosphatidylcholine synthesis, the primary phospholipid in the cell membrane [36]. Choline metabolism alterations are frequently observed under inflammatory conditions [37]. Notably, we observed higher free choline levels in mucosal effusions than in serous effusions (after adjusting for age), which may indicate increased cell membrane breakdown (cytolysis) or enhanced phospholipase activity, which releases free choline and is involved in inflammatory lipid mediator production [37]. This aligns with the notion that mucosal effusion often represents a more intense inflammatory state [8].

The identification of this three-metabolite panel (taurine, glycine, choline) has implications for the objective classification of MEE. Currently, classification relies on the surgeon’s assessment, which can be subjective, even though we employed a standardized inversion test in this study. Moreover, multivariate AROC analysis demonstrated that this panel provided fair discrimination (AAUC = 0.707), independent of age, thereby supporting the concept of “metabolic typing”. This approach, where an objective biochemical signature defines the effusion type, moves beyond simple classification. It could facilitate more precise patient stratification in future clinical trials and aid in developing diagnostic strategies that rely on molecular evidence rather than physical assessment alone, thereby improving standardization in both research and clinical settings.

Nevertheless, the immediate clinical application of this specific NMR-based panel is limited by the relatively large sample volume required for NMR analysis, compared to the small volumes typically retrieved from the middle ear. The primary value of these findings lies in an enhanced understanding of OME pathophysiology. Future research should aim to validate these biomarkers using more sensitive technologies such as mass spectrometry, which requires smaller sample volumes.

This study has some limitations that should be considered when interpreting the findings. First, the study cohort was limited. Due to the low clinical prevalence of mucous effusion in adults and the exclusion of two such samples, we could not implement a full 2 × 2 factorial design (age × viscosity), which prevented a formal analysis of potential interactions between age and effusion type, necessitating the use of ANCOVA to control for age as a covariate. Furthermore, it remains uncertain whether the viscosity-related biomarkers identified here are applicable to rare adult mucous OME instances that may involve distinct etiologies, such as eosinophilic otitis media. Second, the MEE metabolome represents the combined biochemical activities of the host and resident microbiota. Because we did not perform a concurrent microbiome analysis, we could not delineate the specific contributions of microbial metabolism to the observed profiles. Future studies integrating metabolomics and metagenomics would provide a more comprehensive understanding of host-microbe interactions in the middle ear environment. Third, this methodology has technical limitations. While HR-MAS NMR spectroscopy is advantageous for analyzing intact MEE samples with minimal preparation, it offers lower analytical sensitivity than mass spectrometry-based platforms. Consequently, our analysis was restricted to the 31 most abundant metabolites, potentially overlooking lower-concentration signaling molecules or lipid mediators that may play crucial roles in OME pathophysiology. Fourth, potential confounding factors such as the degree of adenoid hypertrophy and variations in conservative treatments (e.g., antibiotics or antihistamines) administered during the pre-operative observation period were not strictly controlled. These factors could potentially influence the inflammatory status and metabolic composition of the effusion. Future studies with strictly controlled prospective designs are needed to address these variables.

## 4. Materials and Methods

### 4.1. Study Population, Sample Collection, and Classification

This study was conducted with the approval of the ethics committee and the institutional review board of our university hospital (IRB No. H-2023-11-009). The participants were prospectively recruited between June 2024 and June 2025. Written informed consent was obtained from all adult participants and the legal guardians of the children involved.

The study cohort comprised children (<12 years of age) and adults (18 years of age) diagnosed with OME. Diagnosis requires MEE confirmation via otomicroscopy or endoscopy. Inclusion was restricted to patients with chronic OME, defined as effusion persisting for at least 3 months, necessitating surgical intervention via myringotomy with ventilation tube insertion.

Stringent exclusion criteria were applied to ensure study population homogeneity. We excluded individuals with known OME predisposing factors, including craniofacial abnormalities (e.g., cleft palate) or syndromes associated with OME (e.g., Down syndrome). Similarly, patients with known immunodeficiencies, pharmacological or intrinsic immunosuppressive conditions, cystic fibrosis, or primary ciliary dyskinesia were excluded. Exclusion criteria comprised a history of other chronic otologic pathologies such as cholesteatoma or chronic mastoiditis, significant anatomical ear defects, or a prior history of skull base malignancy or radiation therapy.

During the surgical procedure, immediately following myringotomy, MEE was aspirated using a Juhn Tym-Tap fluid collection aspirator (Xomed, Jacksonville, FL, USA). The collected fluid was carefully transferred into a sterile 1.5 mL Eppendorf tube.

A standardized objective method was employed to classify effusion viscosity immediately upon collection. The Eppendorf tube containing the MEE was inverted, and fluid movement was observed for 10 s. If the fluid demonstrated downward flow due to gravity within this timeframe, it was classified as “serous.” If the fluid remained stationary at the top of the inverted tube and did not flow downwards within 10 s, it was classified as “mucous” fluid [5].

Overall, 83 MEE samples were collected (Table 2). Following collection and classification, all samples were immediately snap-frozen by liquid nitrogen immersion and stored at −80 °C until HR-MAS NMR analysis.

### 4.2. HR-MAS NMR Spectroscopy

For HR-MAS NMR analysis, each sample was weighed and transferred to a 4 mm zirconia HR-MAS tube (Agilent Technologies, Santa Clara, CA, USA). Subsequently, 20 μL of phosphate buffer (pH 7.4) in deuterium oxide (D_2_O) containing 2 mM 3-(trimethylsilyl)propionic-2,2,3,3-d_4_ acid sodium salt (TSP-d_4_) was added. TSP-d_4_ was used as an internal reference for both chemical shift calibration and metabolite quantification. All reagents were purchased from Sigma-Aldrich (St. Louis, MO, USA).

Spectra were acquired using a 600 MHz Agilent spectrometer equipped with a 4 mm gHX NanoProbe (Agilent Technologies, Santa Clara, CA, USA). The spinning rate was set at 2070 Hz and 298 K, and the spectra were collected using the Carr–Purcell–Meiboom–Gill pulse sequence to suppress broad signals from macromolecules and residual water. The acquisition time, 90-degree pulse (pw), and relaxation delay were set to 3.0 s, 9.6 μs, and 3 s, respectively. Overall, 128 scans were acquired for each sample at a spectral width of 24,038.5 Hz.

### 4.3. Data Processing and Statistical Analysis

All the acquired spectra were manually phased and baseline-corrected. For multivariate statistical analysis, spectra were binned from 0.90 to 8.00 ppm with a bin width of 0.01 ppm, after removing residual water and spinning sidebands. The binned data were normalized using Pareto scaling and analyzed by principal component analysis (PCA) in SIMCA-P+ 12.0 (Umetrics, Umeå, Sweden).

Metabolite identification and quantification were performed using Chenomx NMR Suite 7.1 Professional (Chenomx, Edmonton, AB, Canada) with a 600 MHz metabolite reference library. Metabolite concentrations were further normalized by sample weight and probabilistic quotient normalization to correct for variations in sample amount and overall signal intensity. Univariate statistical analyses, including one-way analysis of variance (ANOVA), analysis of covariance (ANCOVA), and covariate-adjusted receiver operating characteristic analysis (AROC), were performed in R (version 4.0.2; R Foundation for Statistical Computing, Vienna, Austria). All *p*-values were adjusted for multiple comparisons using the Benjamini–Hochberg false discovery rate (FDR), and values less than 0.05 were considered statistically significant.

## 5. Conclusions

This HR-MAS NMR metabolomics study provides strong evidence that patient age is the primary determinant of the metabolic profile of MEE, exerting a greater influence than effusion viscosity. Our findings suggest fundamental differences in the inflammatory and energy metabolism between children and adults with OME, challenging the assumption that viscosity is the main driver of the biochemical environment. Despite the dominant effect of age, we identified taurine, glycine, and choline as age-independent metabolic biomarkers specific to viscosity. These findings offer novel insights into OME heterogeneity and establish a foundation for developing objective metabolic typing, which warrants further validation using high-sensitivity platforms.

## Figures and Tables

**Table 1:** Summary of statistical analyses for metabolites identified in middle ear effusion samples.

Metabolites	ANOVA FDR	Significant Pairwise Comparisons (*p* < 0.05)	Spearman Correlation with Age in Serous Groups	Spearman FDR	ANCOVA FDR (Age-Adjusted)
Pyruvate	2.33 × 10^−8^	SA—MC; SA—SC	0.603	6.73 × 10^−6^	0.5735
Glutamate	1.06 × 10^−6^	MC—SA; SC—SA	−0.433	0.0069	0.3204
Glycine	1.06 × 10^−6^	MC—SA; MC—SC; SC—SA	−0.202	0.1879	0.0139
Choline	2.70 × 10^−5^	MC—SA; MC—SC; SC—SA	−0.258	0.0981	0.0398
Citrate	3.01 × 10^−5^	SA—MC; SA—SC	0.157	0.3276	0.0422
Lactate	3.01 × 10^−5^	SA—MC; SA—SC	0.371	0.0164	0.251
Taurine	7.23 × 10^−5^	MC—SA; MC—SC	−0.263	0.0981	0.0092
Asparagine	0.0002	SA—MC; SA—SC	0.394	0.0158	0.6381
Cystine	0.0002	SA—MC; SA—SC	0.303	0.0571	0.446
Glutamine	0.0003	SA—MC; SA—SC	0.254	0.0981	0.145
Lysine	0.0005	MC—SA; SC—SA	−0.099	0.5524	0.0603
Leucine	0.0009	MC—SA; SC—SA	−0.381	0.0164	0.3763
Serine	0.0009	MC—SA	−0.216	0.1578	0.0888
Glucose	0.0015	SA—MC; SA—SC	0.306	0.0571	0.9772
Betaine	0.0018	SA—MC; SA—SC	0.365	0.0164	0.6033
Succinate	0.0019	MC—SA; MC—SC	−0.253	0.0981	0.1155
sn-Glycero-3-phosphocholine	0.0116	SA—MC; SA—SC	0.071	0.6933	0.3037

FDR-adjusted *p*-values < 0.05 were considered statistically significant. ANOVA, Analysis of Variance; ANCOVA, Analysis of Covariance; FDR, False Discovery Rate; MC, Mucoid; SA, Serous Acute; SC, Serous Chronic.

**Table 2:** Clinical and demographic information of middle ear effusion samples.

Characteristics	Adult	Child	
	Serous	Mucous	Serous
Number of patients (*n*)	45	21	17
Age (years, mean ± SD)	67.71 ± 13.24 (40–86)	3.28 ± 1.90 (1–7)	4.82 ± 3.10 (1–11)
Male:Female	20:25	13:4	15:6

SD, standard deviation.

## Data Availability

The original contributions presented in this study are included in the article/Supplementary Material. Further inquiries can be directed to the corresponding authors.

## Supplementary Materials

The following supporting information can be downloaded at: https://www.mdpi.com/article/10.3390/ijms27010020/s1.

## Abbreviations

The following abbreviations are used in this manuscript:

AAUC	Age-adjusted area under the curve
Ala	Alanine
ANCOVA	Analysis of covariance
ANOVA	Analysis of variance
Arg	Arginine
AROC	Covariate-adjusted receiver operating characteristic
CI	Confidence interval
Cys	Cysteine
FDR	False discovery rate
Gln	Glutamine
Glu	Glutamate
GLM/glm	Generalized linear model
Gly	Glycine
GSH	Glutathione
His	Histidine
HR-MAS	High-resolution magic-angle spinning
HR-MAS NMR	High-resolution magic-angle spinning nuclear magnetic resonance
Ile	Isoleucine
IRB	Institutional review board
Lac	Lactate
Leu	Leucine
Lys	Lysine
MC	Mucous_Child group
MEE	Middle ear effusion
mTOR	Mechanistic target of rapamycin
MUC5B	Mucin 5B
NMR	Nuclear magnetic resonance
OME	Otitis media with effusion
PCA	Principal component analysis
Pro	Proline
ROC	Receiver operating characteristic
SA	Serous_Adult group
SC	Serous_Child group
SD	Standard deviation
Ser	Serine
Tau	Taurine
Val	Valine
